# Recent Advances in Bioorthogonal Ligation and Bioconjugation

**DOI:** 10.1007/s41061-023-00445-6

**Published:** 2023-11-22

**Authors:** Florian M. Zielke, Floris P. J. T. Rutjes

**Affiliations:** Institute for Molecules and Materials, Radboud University, Heyendaalseweg 135, 6525 AJ Nijmegen, The Netherlands

**Keywords:** Bioorthogonal chemistry, Protein functionalization, Transition metals, Bioconjugations

## Abstract

The desire to create biomolecules modified with functionalities that go beyond nature’s toolbox has resulted in the development of biocompatible and selective methodologies and reagents, each with different scope and limitations. In this overview, we highlight recent advances in the field of bioconjugation from 2016 to 2023. First, (metal-mediated) protein functionalization by exploiting the specific reactivity of amino acids will be discussed, followed by novel bioorthogonal reagents for bioconjugation of modified biomolecules.

## Introduction

Bioorthogonal ligations and bioconjugations concern reactions to form bonds, either covalent or well-defined strong non-covalent interactions, between two molecular entities of which at least one is a (modified) biomolecule [1]. The reason that biomolecules are not “just another” entry in a suitable method’s substrate scope is not only the presence of multiple potentially reactive sites, but also the requirement for mild and more stringent reaction conditions compared to those used in small-molecule chemical synthesis. In order to preserve the structure and function of biomolecules, low temperatures (< 37 °C), aqueous media and a near-neutral pH are typically necessary. Furthermore, bioorthogonal ligations and bioconjugations need to proceed rapidly, reaching full conversion within minutes or at most hours at low substrate concentrations. These requirements are lenient in some cases, as smaller biomolecules such as oligopeptides, oligosaccharides, and oligonucleotides may tolerate higher temperatures, for example, or organic co-solvents.

In recent years, bioorthogonal ligations and bioconjugations have seen a steady increase in publications reporting new reactions, reagents and applications [2]. This development was reflected in awarding the 2022 Nobel Prize in Chemistry jointly to Carolyn R. Bertozzi, Morten Meldal and K. Barry Sharpless for their contributions to the development of click chemistry and bioorthogonal chemistry.

Bioconjugation methods will differ from one biomolecule to another, and a particular method may not be suitable for every biomolecule, even if they are from a similar class. Nguyen and Prescher recently highlighted that there is no perfect bioorthogonal reaction [3]. The “best fit” for a specific application has to be chosen instead. In this review, we will focus on recent advances in bioorthogonal ligations and bioconjugation, highlighting new reagents and novel methods developed between 2016 and 2023. First, bioconjugation reactions of natural amino acids will be described, followed by bioconjugation to modified biomolecules.

## Amino Acid Functionalization

Bioconjugation of synthetic molecules to unfunctionalized proteins and enzymes exploits the unique reactivity of amino acids based on their functional groups, local environment (buried inside protein vs exposed on protein surface) and natural abundance. In this section, heteroatom arylations, transition metal-mediated C–H functionalization, and new organic reagents for N-terminus and amino acid-specific labeling will be highlighted. The topic of metal-mediated bioconjugations has been reviewed in greater detail by Ohata et al. [4] and Zhang et al. [5].

### Metal-Mediated Functionalization

#### Cysteine Arylations

Cysteine is one of the most commonly targeted amino acids for bioconjugation. In aqueous buffer, the thiol group of cysteine is more acidic than other nucleophiles such as alcohols or primary amines [6]. Additionally, sulfur is more polarizable than oxygen or nitrogen. This accounts for an overall higher reactivity towards electrophiles than other protein nucleophiles. Cysteine in this respect also benefits from its relatively low abundance in the human proteome [7].

**Fig. 1:**
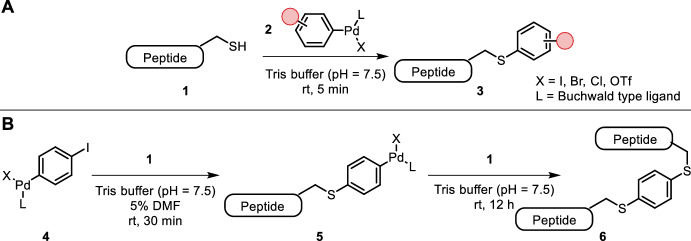
**A** Oxidative addition complexes of aryl halides with palladium-Buchwald-ligand complex **2** yield cysteine arylation products within minutes; **B** dihaloarenes used for tandem cysteine arylation

Many metal-catalyzed reactions with biological substrates suffer from high metal loadings due to unspecific and unproductive coordination. Rojas et al. showed that oxidative addition complexes of a water-soluble palladium-Buchwald ligand complex (**2**) with a desired aryl halide (Fig. 1A) underwent cysteine-selective arylation in an aqueous buffer (pH = 7.5) at room temperature within minutes, thereby enabling rapid bioconjugation of functional handles, fluorophores, affinity tags, cross-linkers and antibody drug conjugates [8, 9]. Supposedly, the interaction between the soft thiol nucleophile and the aryl palladium(II) species guided the selectivity for cysteine over other amino acids. Importantly, the palladium complexes were compatible with tris(2-carboxyethyl)phosphine (TCEP), which is commonly used for reduction of disulfide bonds. A complementary approach from the same group used unprotected aryl-halide-containing proteins without cysteine residues to form oxidative addition complexes with palladium. The addition of a thiol nucleophile including cysteine-containing peptides also led to thiol arylation products [10]. The broad utility of this method was highlighted by the site-selective coupling of arylated oligonucleotides to cysteine-containing proteins [11]. Another example concerned the incorporation of an electrophilic trap into the aryl group to capture another nucleophile, such as lysine, after palladium-mediated cysteine arylation, resulting in stapling of proteins or cross-linking of two interacting proteins [12].

The same group achieved bioconjugation of two cysteine-containing peptides using this chemistry in tandem (Fig. 1B). Mono-oxidative addition product **4** afforded cysteine arylation and regeneration of a palladium(0) species that formed another oxidative addition product with the cysteine-coupled haloarene (**5**). The addition of a second cysteine-containing protein then afforded the conjugated product (**6**) [13]. Alternatively, the addition of a ^11^CN radiolabel (using Na^11^CN) yielded radiolabeled proteins [14]. This type of radiolabeling could be carried out in one pot.

**Fig. 2:**
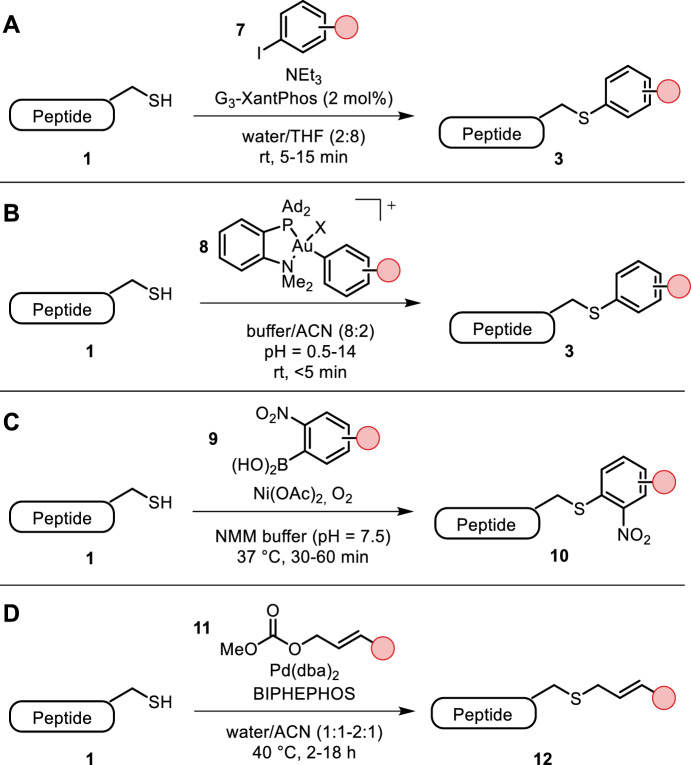
**A** Catalytic cysteine arylation and alkynylation with G_3_-XantPhos; **B** gold complexes used for cysteine arylation proceeding faster and over a wider pH range than their palladium counterparts; **C** Chan-Lam coupling of 2-nitroaryl boronic acids for selective cysteine arylation; **D** Tsuij-Trost allylation conditions forge a naturally occurring allyl thioether linkage

Catalytic cysteine arylation and alkynylation were reported by Al-Shuaeeb et al. in a cross-coupling-like manner using catalytic amounts of G_3_-XantPhos-Pd and aryl/alkynyl halides (**7**) [15]. The reaction proceeded at room temperature in water with significant amounts of THF as co-solvent within a few minutes, albeit with the addition of triethylamine required. Short peptides were rapidly diversified, but larger biomolecular targets required larger amounts of palladium (Fig. 2A).

Messina et al. and Kung et al. independently described cysteine-selective arylations using gold complexes [16, 17]. In addition to the benefits of palladium-mediated arylation, gold arylations using complex **8** worked within a wider pH range (pH = 0.5–14) and displayed faster kinetics than their palladium-based counterparts, outperforming them by a kinetic factor of approximately 9 (Fig. 2B). On the other hand, these reactions required the addition of 20% ACN as co-solvent. The authors introduced variously functionalized aryl groups to short peptides including drugs, dyes and affinity tags. Gold-mediated cysteine and thio-sugar arylations were used in ^18^F labeling by Mc Daniel et al. [18].

A Chan-Lam approach to cysteine arylation was reported by Miller et al. Cysteine-selective arylation of 2-nitro-substituted aryl boronic acids **9** was achieved in aqueous buffer and completed within 1 h (pH = 7.5, 37 °C, Fig. 2C). Interestingly, the 2-nitro substitution was found to be crucial for the reaction progress, allowing orthogonal functionalization with boronic acids [19–21].

Schlatzer et al. performed chemoselective cysteine allylations using a palladium-mediated Tsuij-Trost protocol at elevated temperatures (40 °C) with ACN as co-solvent (Fig. 2D) [22]. Allyl thioether linkages (**12**) occur naturally in cysteine prenylation reactions. Using this chemistry, the authors introduced various labels (biotin, fluorescent) and bioorthogonal functional groups (azide, alkyne) to short peptides and highlighted the applicability of their chemistry by labeling Hsp-27 and UBL3.

#### Lysine and *N*-Arylations

In contrast to cysteine, lysine is one of the more abundant amino acids in the human proteome. Proteins usually have more than one solvent-exposed lysine residue, making selective lysine functionalization challenging, but opening the door for universal methods. The protonated lysine amino group is less acidic than the N-terminal amine. Selective lysine modifications can therefore be achieved at high/neutral pH, and selective N-terminus modifications can be achieved at low pH [23].

Expanding on their cysteine arylation work, Lee et al. used similar palladium oxidative addition complexes for lysine arylations in DMSO at room temperature [24]. Reaction progress was slower, with full conversions obtained within hours. Oxygen- and nitrogen-based nucleophiles of other amino acids were tolerated with the exception of primary amides and guanidine functional groups, where some competitive reactivity was observed. Similarly, an unprotected N-terminus or C-terminal amide gave side reactivity. At the cost of overall yield, these side reactions could be suppressed by employing the palladium complex as the limiting reagent. Importantly, the method appeared incompatible with the thiol function of cysteine residues, reacting faster than lysine under the reported conditions.

**Fig. 3:**
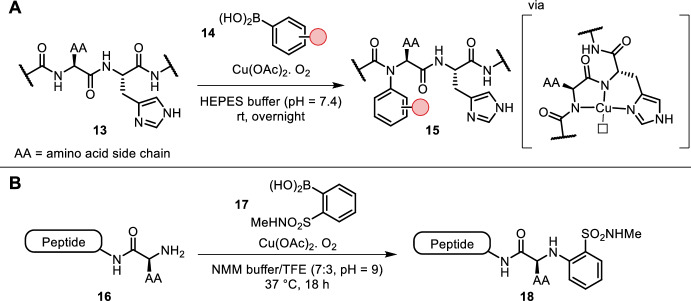
**A** Directed backbone *N*-arylation by mimicking the ATCUN motif; **B** ortho-sulfonamide arylboronic acids used for selective N-terminal arylation

Ohata et al. described a histidine-directed backbone amide arylation/alkenylation under Chan-Lam conditions with copper(II) in aqueous buffer (pH = 7.4) at room temperature (Fig. 3A) [25]. The peptide coordination to copper is thought to mimic the well-known amino terminal copper and nickel (ATCUN) motif. Within the peptide substrate a tridentate complex could be formed, which has one free coordination site (compared to the ATCUN tetradentate) for transmetallation of the boron species. While reaction times were long (overnight), this approach enabled site-selective backbone amide functionalization. An amino acid combination of N-terminal pyroglutamate-histidine provided faster reaction rates yielding arylation and alkenylation products within minutes [26]. This new tag is canonically encoded, easing the introduction of this reaction into the bioorthogonal toolkit. Miller et al. expanded their Chan-Lam-type chemistry to selective N-terminus arylation using ortho-sulfonamide-substituted boronic acids (Fig. 3B, 17) [27]. While some reaction progress was observed in a strictly aqueous solvent, the addition of organic co-solvents improved the yields dramatically. Bioorthogonal groups can be easily introduced at the sulfonamide functional handle. Furthermore, the authors found only ortho-substituted sulfones, sulfonamides and halogens to mediate product formation.

#### Tryptophan C–H Functionalization

**Fig. 4:**
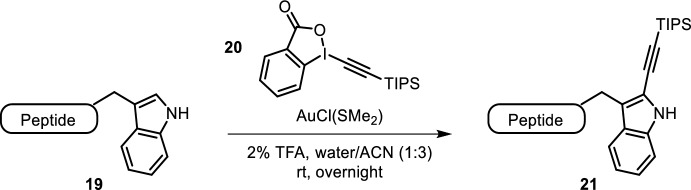
C-2-selective tryptophan alkynylation with hypervalent iodide reagent **20** using gold catalysis

The indole-containing amino acid tryptophan can be selectively engaged in metal-mediated cross-couplings at the 2-position. Previous metallocarbene-based modifications were tryptophan-selective, but yielded both N- and C-modified products [28]. Tolnai et al. expanded their previous indole alkynylation protocol using hypervalent iodide reagent **20** to carbon-selective ethynylation of tryptophan in dipeptides [29]. The method proved to be chemoselective for tryptophan even in the presence of tyrosine, phenylalanine, serine and lysine residues. The reaction, however, required ACN, slightly elevated temperatures (40 °C) and long reaction times (up to 24 h) to reach full conversion, even though significant conversion was typically already observed within 1 h. Hansen et al. showed that longer peptides were also suitable substrates for this chemistry (Fig. 4) [30]. They further demonstrated successful protodesilylation of alkynylated melittin, followed by copper-catalyzed alkyne-azide cycloaddition (CuAAC) with a fluorescently labeled azide.

**Fig. 5:**
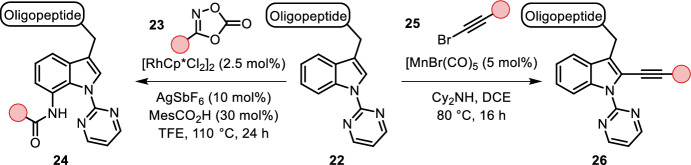
C-2- or C-7-selective C–H functionalization of *N*-(2-pyrimidyl)tryptophan under manganese or rhodium catalysis

Ruan et al. and Wang et al. found a 2-pyrimidyl-directing group that enabled both C-2-selective alkynylation using a manganese(I) catalyst system and C-7-selective amidation under rhodium(III) catalysis, opening the space for rapid difunctionalization of tryptophan (Fig. 5). The reliance on a directing group, non-aqueous solvents (DCE and TFE) and high temperatures (80 and 110 °C), however, limit the scope to small peptides [31, 32].

### Developments in Organic Reagents

#### Targeting the N-Terminus

The protein amino terminus presents a unique opportunity for bioconjugation for general reactivity, but also targeted reactivity depending on the particular N-terminal amino acid. N-terminus-specific bioconjugation has been reviewed in greater detail by De Rosa et al. [33], Rosen and Francis [34] and Tantipanjaporn and Wong [35].

##### General Methods

**Fig. 6:**
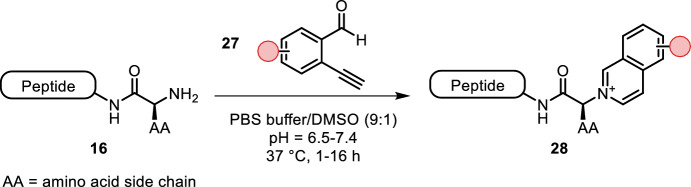
Condensation of **27** with the N-terminus followed by spontaneous 6-endo-dig-cyclization yielding isoquinolinium adducts

Deng et al. used ortho-ethynylbenzaldehyde derivatives **27**, which upon condensation with the amino terminus underwent a 6-endo-dig-cyclization, yielding the corresponding isoquinolinium adducts (Fig. 6) [36]. The reaction required DMSO as an organic co-solvent, was carried out at 37 °C and finished in hours. As expected, N-terminus over lysine selectivity increased at lower pH (pH = 6.5), but faster conversion was observed at higher pH (> 7.4). Moderate to excellent N-terminal selectivity was observed for all N-terminal amino acids with the exception of proline, as the secondary amine cannot undergo cyclization. Various bioorthogonal functional groups were tolerated, and the utility of the reaction was demonstrated by introduction of a bioorthogonal handle and fluorescent labeling of lysozyme and RNase A.

##### Amino Acid-Specific Methods

*Cysteine* The topic of N-terminal cysteine bioconjugation is reviewed in greater detail by Lee [37].

Dempsey et al. and Hawala et al. both independently reported methods for native chemical ligation of N-terminal cysteines using thioesters [38, 39]. Transesterification with thiol followed by intramolecular *S*–*N* acyl transfer yielded the desired native chemical ligation products. Both groups made use of a mercaptoethanesulfonate thioester as transesterification agent, while experiments were performed in an aqueous buffer at room temperature and at close to neutral pH (pH = 7.0 and 7.4, respectively). Reactions times were a few hours and no acylation of internal cysteine or lysine residues was observed. Cole’s group showed that suitable thioester precursors can be easily obtained from widely used NHS esters.

**Fig. 7:**
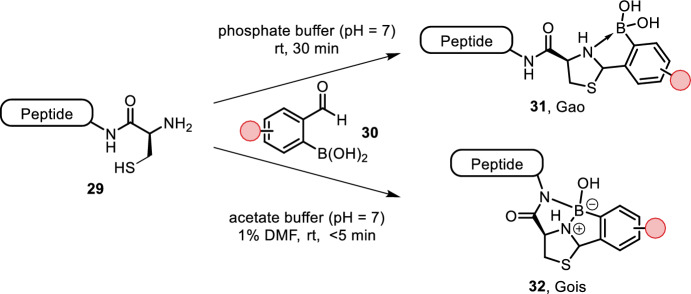
N–B coordination accelerates thiazolidine formation of benzaldehyde derivatives with N-terminal cysteines

Condensation of an aldehyde with an N-terminal cysteine yields a thiazolidine adduct. Formation of this adduct, however, requires acidic conditions and suffers from slow kinetics. Bandyopadhyay et al. and Faustino et al. both reported increased rates using ortho-boronic acid-substituted benzaldehydes (Fig. 7, 30) [40, 41]. The thiazolidine adduct was stabilized by nitrogen coordination to boron’s empty p-orbital. This modification allowed for fast reaction rates (2.5–5.5 × 10^3^ M^−1^ s^−1^) at neutral pH (pH = 7) and room temperature. Interestingly, the two groups proposed slightly different adducts. Adduct formation is reversible by either addition of hydroxylamines or slightly acidic conditions. The utility of this method was demonstrated by fluorescent labeling of the villin headpiece among other short peptides.

**Fig. 8:**
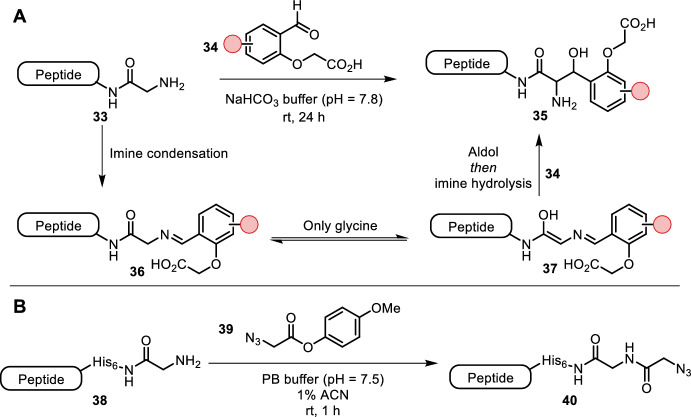
**A** Aldol addition of N-terminal glycine to benzaldehyde derivative **35**. Keto-enol tautomerism is only found for glycine. **B** Azide **39** selectively acylates N-terminal glycine with a His-tag

*Glycine* Purushottam et al. found that N-terminal glycine reacted with substituted benzaldehydes **34** in an aldol fashion (Fig. 8A) [42]. The reaction proceeded in slightly basic aqueous buffer (pH = 7.8) at room temperature, while reaction times were long, taking up to 24 h. The ortho-substituted acid was found to be crucial for this unique reactivity. The authors proposed that the carboxylic acid acts as a hydrogen bond acceptor during the reaction. The excellent utility and selectivity of the reaction was demonstrated by fluorophore labeling of SUMO1 in a cell lysate.

Martos-Maldonado et al. reported an N-terminal glycine His-tag-specific terminal *N*-acylation (Fig. 8B) [43]. Full product formation was observed, with para-methoxyphenyl methyl ester **39** successfully installing an azide handle in aqueous buffer (pH = 7.5) at room temperature within a few hours. Without the His-tag, no reaction progress was observed. Mechanistic investigations showed that histidine assists in deprotonation of the initially formed tetrahedral intermediate rather than in situ formation of a transient acyl imidazole species. Biotin and fluorescent labeling of multiple His-tagged proteins (BIR2 and EGFP) highlighted the applicability of this method.

#### Amino Acid-Specific Labeling

##### Tyrosine

Choi et al. reported a chemoselective tyrosine sulfur fluoride exchange (SuFEx) reaction with aryl fluorosulfates [44]. They used model substrates of other amino acid nucleophilic sites showing no reactivity towards the SuFEx reagent even at elongated exposure times (12 h), with the exception of indole showing slight product formation. Reaction of a tyrosine model substrate was significantly faster (> 90% yield after 1.5 h). The utility of this methodology was demonstrated by fluorescent labeling of cell-penetrating peptide TAT 47–57 in DMSO at room temperature and PEGylation of recombinant human erythropoietin in basic aqueous buffer (pH = 8). The authors found that efficient biaryl sulfate formation required the addition of tetramethyl guanidine base.

**Fig. 9:**
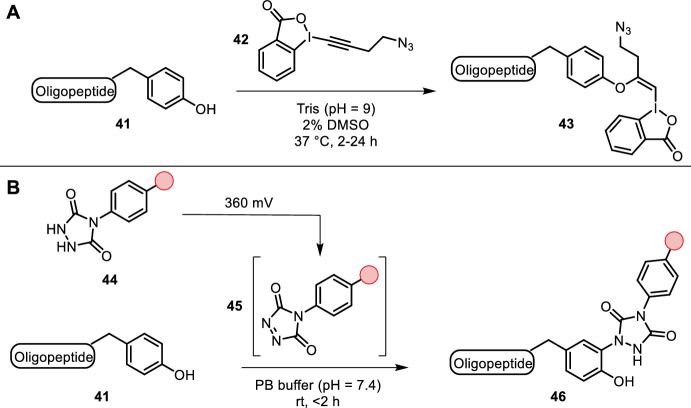
**A** Tyrosine-selective functionalization with hypervalent iodine **42** yields two functional handles for further conjugation. **B** Electrochemical in situ oxidation of urazoles to PTAD enable tyrosine-selective labeling

Declas et al. and Tessier et al. found hypervalent iodine reagent **42** to facilitate chemoselective addition of tyrosine residues to the alkyne (Fig. 9A) [45, 46]. The reaction proceeded in basic aqueous buffer (pH = 9) with a minute amount of organic co-solvent (2% DMSO). The resulting species possessed two functional handles for further modification, the azide and the hypervalent vinyl iodide. The latter could be engaged in metal-mediated cross-couplings. Multiple oligopeptides appeared to be suitable substrates and all amino acids with the exception of cysteine were tolerated, which itself underwent efficient bioconjugation. Antibodies and proteins were functionalized using this strategy, albeit in lower overall conversion.

Alvarez-Dorta et al. reported an in situ electrochemical oxidation of urazoles **44** to 4-phenyl-3*H*-1,2,4-triazole-3,5(4*H*)-diones (PTAD, **45**) for tyrosine-specific labeling in an ene-like fashion (Fig. 9B) [47]. The reaction proceeds in aqueous buffer at room temperature within hours and was found to be selective for tyrosine residues. This is superior to previously employed methods, as PTAD anchors have been shown to yield undesired lysine labeling due to decomposition into isocyanates in the presence of water. Importantly, potential of 360 mV was required to only oxidize urazoles without affecting other oxidizable amino acids such as tyrosine, phenylalanine, lysine, tryptophan and histidine. Furthermore, electrochemical oxidation of urazoles is not only milder compared to chemical oxidants, which may not be suitable for sensitive substrates, but also more effective. More than double the amount of tyrosine residues are modified in bovine serum albumin (BSA) using electrochemical oxidation compared to chemical oxidants under identical reaction conditions (average number of 9.1 vs 3.7). The authors showed the versatility of this method by labeling oxytocin, angiotensin 2, BSA, epratuzumab and glucose oxidase, which fully retained their enzymatic activity after tyrosine labeling, thereby highlighting the mildness of their oxidation methodology.

##### Lysine

Lysine bioconjugation methods were recently reviewed in greater detail by Haque et al. [48] and Tantipanjaporn and Wong [35].

**Fig. 10:**
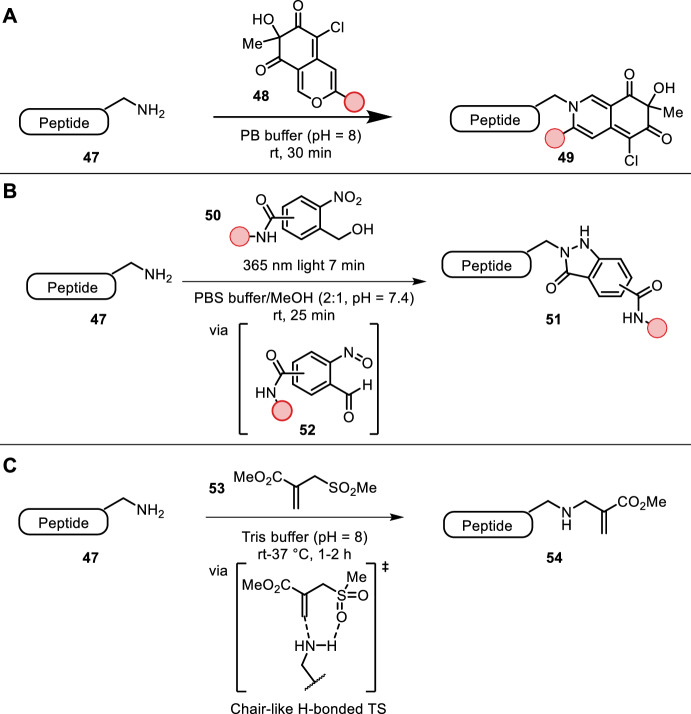
**A** Primary amine-selective azaphilone derivatives yield γ-pyridones; **B** photoactivation of nitro-benzyl alcohols yield nitroso intermediate **52** forming an indazolone adduct with primary amine nucleophiles; **C** regio- and chemoselective lysine bioconjugation with acrylate **53**. Lysine selectivity originates from a chair-like H-bonded transition state

The natural product class azaphilones (**48**), a unique family of fungal polyketide metabolites, were found to readily react with primary amines under physiological conditions, forming vinylogous γ-pyridones stable to acid and base. Yi et al. showed that all primary amines (lysine and N-terminal) were efficiently labeled in proteins and antibodies (Fig. 10A) [49]. The reaction proceeded in aqueous buffer at room temperature within minutes for small peptides. Even faster reaction rates were obtained in slightly basic medium. Successful fluorescent labeling of lysozyme, RNase A, histone 2A and HER2 antibody was achieved. An additional advantage of this type of natural product is its inherent fluorescent properties. The authors reported a modular synthesis of vinylogous γ-pyridones, allowing for straightforward incorporation of the desired bioorthogonal tags.

Photoactivation (365 nm) of ortho-nitrobenzyl alcohols **50** resulted in aryl nitroso aldehydes **51**, which could react with a primary amine (e.g. lysine), forming an indazolone adduct as described by Guo et al. (Fig. 10B) [50]. Full conversion was obtained within 30 min in aqueous buffer (pH = 7.4) with methanol as co-solvent at room temperature. Surprisingly, the nitroso intermediate was found to be relatively stable in neutral buffer, with half times of > 20 h. An acyl functional group on the aromatic ring allowed for introduction of various bioorthogonal handles. Small peptides were successfully functionalized/macrocylized. This method was used for live cell imaging of HER2 and protein kinases.

Matos et al. reported sulfonyl acrylate reagent **53** for use in chemo- and regioselective lysine labeling (Fig. 10C) [51]. The resulting acrylate functional handle was used in subsequent Michael-type reactions. Lysine labeling proceeded in slightly basic aqueous buffer (pH = 8) at room temperature within an hour. Chemoselectivity was driven by a transient hydrogen bond between the amine and sulfonyl groups in a chair-like aza-Michael-type transition state, decreasing the activation barrier and increasing lysine nucleophilicity, thereby significantly increasing the reaction rate and regioselectivity. Regioselective outcomes could be predicted by computational studies under the hypothesis that the most acidic reagent-accessible lysine would be the most reactive towards sulfonyl reagent **53**. Cysteine residues were unaffected by this method.

**Fig. 11:**
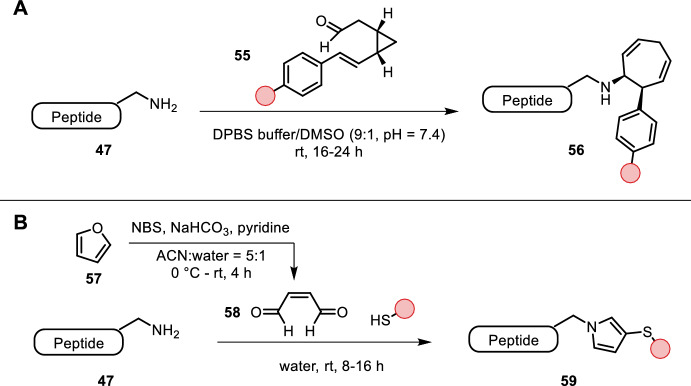
**A** Condensation of primary amines with vinyl cyclopropyl aldehyde **55** triggers a Cope-rearrangement, making the condensation irreversible. **B** FuTine reaction between **58** and a thiol and amine nucleophile forges the formation of the substituted pyrrole **59**

Apel et al. reported a lysine-specific ligation using vinyl cyclopropyl aldehyde **55**, which upon condensation with the lysine amino group triggered a Cope rearrangement to a cycloheptadiene, rendering the condensation irreversible (Fig. 11A) [52]. The reaction was carried out in aqueous buffer with 10% DMSO as co-solvent at room temperature. The modular synthesis of vinyl cyclopropyl aldehyde **55** allowed for the introduction of (bioorthogonal) functional handles via a Suzuki–Miyaura coupling. The excellent chemoselectivity was demonstrated by a labeling study of EGFP.

Recently Wang et al. reported a bioinspired multicomponent furan-thiol-amine (FuTine) reaction for lysine labeling (Fig. 11B) [53]. Oxidation of furan to *cis*-2-butene-1,4-dial (BDA, **58**) followed by addition of a thiol and amine nucleophile forged the formation of 3-thio-*N*-substituted pyrroles in one pot. The more nucleophilic thiol is added in a 1,4-fashion first, followed by imine formation, after which rearrangement and aromatization yield the pyrrole product. The multicomponent reaction could be carried out in aqueous buffer at room temperature over several hours. The authors were able to modify both lysine and cysteine residues with various functional handles, as well as performing peptide macrocyclization and stapling. The applicability of this method was highlighted by labeling of multiple proteins even in complex cell lysate mixtures. One drawback of their method is the low stability of **58**, which needs to be freshly prepared before each labeling experiment.

##### Cysteine

The topic of cysteine bioconjugation using organic reagents was reviewed in greater detail by Chen and Gao [54].

**Fig. 12:**
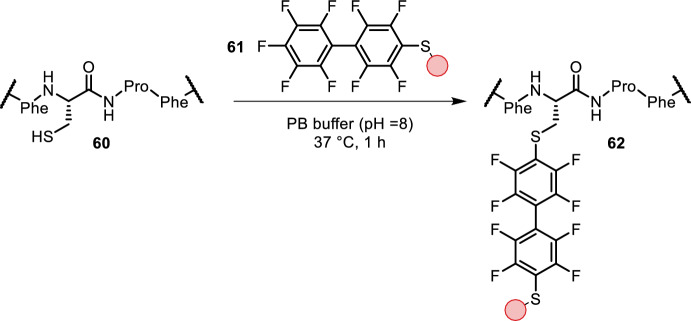
π-Clamp cysteine arylation using perfluoro reagent **61** in an S_N_Ar reaction

Zhang et al., Dai et al. and Dai et al. found a new tag (Phe-Cys-Pro-Phe, **60**) for site-selective cysteine perfluoroarylation, which they termed the “π-clamp” (Fig. 12) [55]. The reaction rate was 0.76 M^−1^ s^−1^ and required slightly basic aqueous buffer (pH = 8) at 37 °C. Site selectivity was demonstrated by appending the heavy chains of antibodies by this tag. Reaction with perfluorobiaryl reagent **61** only arylated the π-clamp cysteines. Solid-state nuclear magnetic resonance (NMR) suggested that the phenylalanine side chains interact with perfluorobiaryl reagent **61**. Further DFT calculations suggested that the π-clamp promotes the reaction by lowering the activation energy and generating a more stable product [56]. The addition of simple inorganic salts significantly affected the reaction rate, most likely by modulating the specific hydrophobic interactions between the π-clamp and perfluorobiaryl reagent **61** [57]. Using this chemistry, the authors were able to ligate peptides, biotin, fluorescent and PEG probes as well as bioorthogonal alkyne handles to peptides bearing a π-clamp. Both N- and C-terminal and middle positioning of the π-clamp tag were tolerated.

**Fig. 13:**
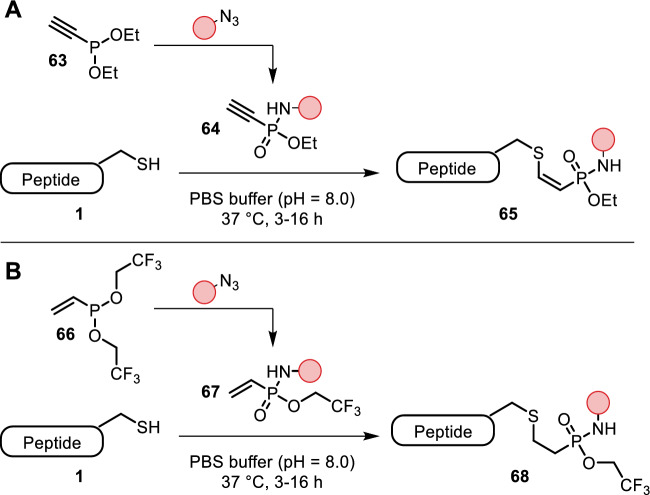
**A** Staudinger phosphonite reaction between **63** and an azide yields electrophilic phosphoramidate **64** for cysteine-selective bioconjugation; **B** similarly, Staudinger phosphonite reaction with vinyl phosphonite **66** yields **67** for cysteine-selective bioconjugation

Building on previous accomplishments of the Staudinger phosphonite reaction [58], Kasper et al. expanded their bioorthogonal chemistry to cysteine-selective bioconjugation [59]. A chemoselective Staudinger phosphonite reaction between ethynylphosphonite **63** and an azide yielded ethynylphosphonamidate **64**. The authors noticed that accompanied with this transformation is a change in electronic properties of the ethynyl substituent from electron-rich to electron-poor, which facilitated subsequent chemoselective addition of cysteine residues forging a stable adduct with *Z*-selectivity. The reaction proceeded in slightly basic aqueous buffer (pH = 8) at 37 °C (Fig. 13A). The adducts did not undergo retro-Michael reactions when exposed to external thiols, which is an advantage over maleimide conjugates. Applications were demonstrated by conjugation of fluorophores to antibodies and GFP to cyclic cell-penetrating peptides.

The very same group followed up their work with vinylphosphonites [60]. Similar to the ethynylphosphonite, they were reacted with azides in a Staudinger phosphonite reaction (**66**–**67**), followed by cysteine addition based on the change in electronic properties of the vinyl substituent (Fig. 13B). Sufficient thiol addition and reaction rates were only obtained with additional electron-withdrawing *O*-substituents, identifying the *O*-trifluoroethyl substituent as the most reactive one. Just as their unsaturated analogs, the cysteine adducts were stable towards retro-Michael additions and retained their cysteine selectivity. Successful conjugation of fluorophores to antibodies showcased their application in bioorthogonal chemistry.

**Fig. 14:**
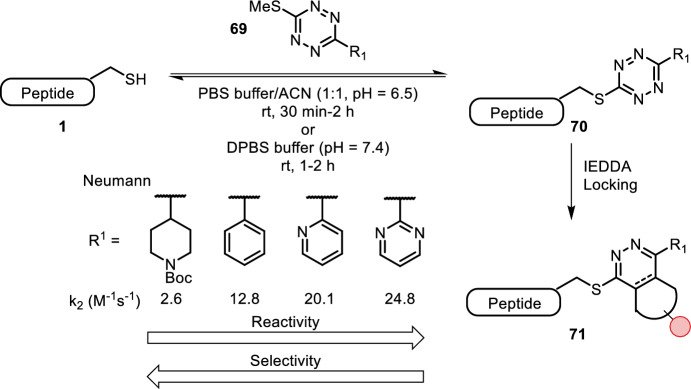
Reversible TeTEx between a cysteine-containing peptide and thiomethyl tetrazine **69**, followed by IEDDA locking with strained alkynes or TCOs

Gavriel et al. and Tallon et al. both independently reported the use of asymmetric thiomethyl tetrazines **69** in a reversible tetrazine-thiol exchange reaction (TeTEx) for cysteine bioconjugation (Fig. 14) [61, 62]. Subsequent inverse-electron-demand Diels–Alder (IEDDA) reaction rendered the thiol exchange irreversible. TeTEx was carried out in aqueous buffer (pH = 7.4) at room temperature. The reaction was cysteine-selective and tolerated nucleophilic sites of other amino acids. Neumann noticed that more electron-deficient tetrazines reacted faster (in general reaction rates of 1–100 M^−1^ s^−1^ were observed depending on tetrazine substitution), but also led to small amounts of byproducts presumably by reaction with nucleophilic sites of other amino acids. Both groups showed cysteine-selective labeling of short peptides and an IEDDA reaction with strained alkynes or *trans*-cyclooctenes (TCOs) rendering the thiol exchange irreversible (locking). Fox highlighted the applicability of this method by cysteine conjugation to GFP followed by locking with a simple TCO. Other examples concerned fluorescent HeLA lysate labeling and live cell fluorescence and biotin labeling.

## Bioconjugations Using Bioorthogonal Reactions

In this section we will focus on bioconjugations with previously modified biomolecules already bearing a bioorthogonal functional group, thereby shifting the focus from target-based approaches to reagents and their applications. The discussion will most prominently feature phosphine-based reagents for Staudinger reactions and phospha-Michael reactions, boron-based bioconjugations, new cyclic alkynes for strain-promoted cycloadditions and the duality of tetrazine in fluorogenic reactions.

### Phosphine-Based Reagents

The history and development of bioorthogonal phosphine reagents was carefully reviewed by Prescher [63].

#### Staudinger Ligation

Pioneering work by Saxon and Bertozzi in the area of Staudinger-type ligations involved an electrophilic ester trap on an arylphosphine [64]. Reaction of this phosphine with an azide initially provided an unstable iminophosphorane, which intramolecularly attacked the ester forming a cyclic intermediate, which was then hydrolyzed to yield a stable bioconjugated amide.

**Fig. 15:**
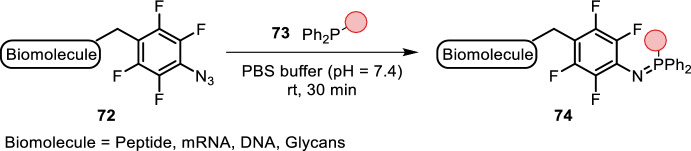
Staudinger ligation using PFAAs yields stable iminophosphorane adducts

Mechanistic studies suggested that reaction rate enhancement could be achieved by increasing either the phosphine nucleophilicity or azide electrophilicity [65, 66]. The latter seemed a more feasible approach, as electron-rich phosphines are typically more prone to oxidation [67]. Inspired by this, Sundhoro developed perfluoroaryl azides **72** (PFAAs), providing iminophosphorane products that were stable towards hydrolysis and aza-phosphonium ylide reactions (Fig. 15) [68]. Their potential has been proven by efficient labeling of cell-surface glycans [68], proteins [69], mRNA [69] and DNA [69]. PFAA handles have also found use in glucose imaging in vivo due to their enhanced rate of reactivity compared to other glucose-derived azides. Efficient labeling was carried out in aqueous buffer (pH = 7.4) within 30 min [70]. Similar to PFAAs, 2,6-dichlorophenyl azides, reported by Meguro, provided hydrolysis- and oxidation-stable iminophosphorane adducts as well. Their utility and bioorthogonality was demonstrated in fluorescent labeling of GST-HaloTag protein living cells [71].

##### Light-Triggerable

**Fig. 16:**
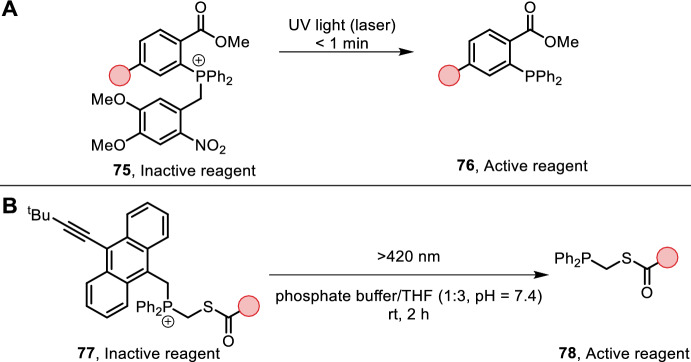
**A** Photo-uncaging of a Staudinger ligation reagent. NB: the authors only apply this technique in various biological settings (cells, zebrafish) with specific reaction conditions; **B** photo-uncaging of a traceless Staudinger ligation reagent

The Staudinger reaction was rendered visible-light-triggerable by Shah et al. through implementation of photocages. Ortho-nitrobenzylphosphonium salts 75 could release the active Staudinger ligation reagent (**76**) under irradiation of UV light (Fig. 16A) [72]. Successful Staudinger ligation has been observed in vitro and in vivo (in zebrafish) with metabolically labeled azidosialic acid, highlighting the applicability of this technique in biologically relevant settings.

Moving away from UV light activation, as this can be damaging for organisms, the use of an anthracene-based photocage was reported in a traceless Staudinger ligation with blue light by Hu et al. (Fig. 16B) [73]. The traceless variation of the Staudinger ligation forges the formation of amide bonds and ligation products that do not contain unnatural phosphine oxide derivatives. A few anthracene-based analogs were synthesized identifying compound **77** with improved photolytic properties [74]. The authors demonstrated small peptide ligation in aqueous buffer (pH = 7.4) with THF as co-solvent at room temperature. Notably, irradiation times were long, up to 2 h, with ligation taking 16 h.

#### Cyclopropenones in Bioconjugations

**Fig. 17:**
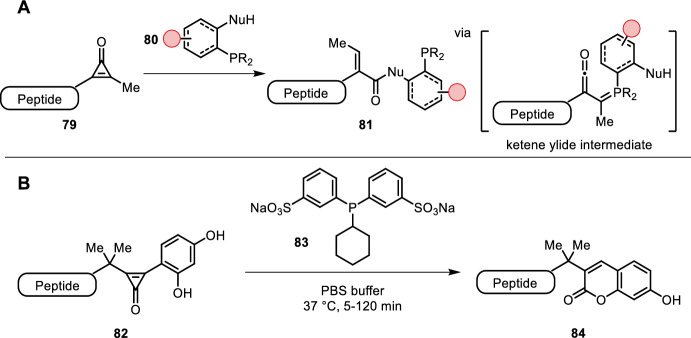
**A** Disubstituted cyclopropenones used in bioconjugation with nucleophilic phosphines. The ketene ylide intermediate is trapped by a nucleophile, yielding ring-opened adducts; **B** cyclopropenone **34** yielding a fluorescent coumarin moiety upon treatment with a water-soluble phosphine

Triarylphosphines have proven themselves in bioorthogonal reactions, but coupling partners beyond azides have also been identified. Row et al. found disubstituted cyclopropenones (CpOs, e.g. **79**) sufficiently stable in biological contexts and explored their use in bioconjugation [75]. CpOs possess significant ring strain that can be leveraged by phospha-Michael addition reactions. Conjugate addition of the phosphine was followed by ring opening yielding a ketene ylide, which could then be trapped with a nucleophile (Fig. 17A) [76]. Therefore, in contrast to Staudinger reagents, CpO reagents bear an ortho-nucleophilic trap (**80**) [75]. Modifying the cyclopropenone to a thiocarbonyl provided increased reaction rates, attributed to lower LUMO (lowest unoccupied molecular orbital) energies. They also showed similar stability under physiological conditions [77]. Prescher demonstrated the utility of CpOs by biotin labeling of a CpO-modified GFP [75] and covalent biomolecule cross-linking of the split NanoLuc [78] in aqueous buffer (pH = 7.4) at 37 °C. Furthermore, they developed a fluorescent turn-on probe (**82**), which upon treatment with a water-soluble phosphine (**83**) yielded a coumarin product (**84**) that could be used for live imaging in cells (Fig. 17B). Of importance is the gem-dimethyl substitution, which directs a regioselective phosphine addition onto the cyclopropenone. Using this technique in combination with bioorthogonal tetrazine/*trans*-cyclooctene cycloadditions enabled dual metabolite real-time imaging with no washing steps [79].

### Boron-Based Reagents

Boron-based bioconjugations are reviewed in more detail by Gois [80].

**Fig. 18:**
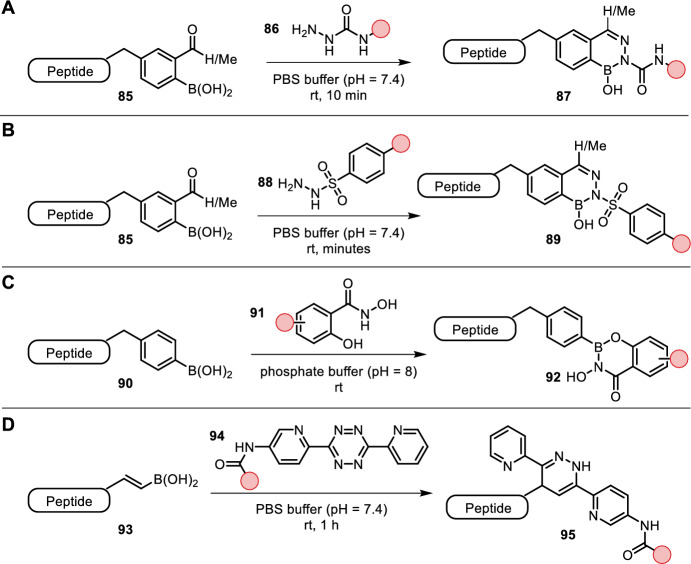
**A** Ortho-acyl arylboronic acids form stable diazaborine adducts upon treatment with semicarbazides; **B** ortho-acyl arylboronic acids from stable diazaborine adducts upon treatment with sulfonyl hydrazides **C** arylboronic acids treated with salicylhydroxamate **91** form adducts that are cleaved upon acidification; **D** vinylboronic acids react with tetrazines in an IEDDA reaction, 2-pyridyl substitution accelerates the reaction by coordination to boron’s empty p-orbital

Previously discussed arylboronic acids cannot only be used for (metal-mediated) amino acid or N-terminus bioconjugations, but can additionally be used for bioconjugation with semicarbazides **86** as reported by Bandyopadhyay et al. and Cambray et al. (Fig. 18A) [81, 82]. They found rates of semicarbazone conjugation to be greatly increased by the ortho-boronic acid (**85**) forming a stable diazaborine product. The condensation proceeded in aqueous buffer (pH = 7.4) at room temperature within minutes. The promising applicability of this chemistry was demonstrated by fluorogenic labeling of bacterial cell walls in blood serum. They successfully synthesized semisynthetic amino acids containing either a semicarbazide or ortho-acyl arylboronic acid. Both unnatural amino acids appeared to readily incorporate into cell walls of bacteria and could subsequently be labeled with a suitable fluorescent dye-containing reagent.

Semicarbazide condensation, however, suffers from low conversions at low micromolar concentrations. Chowdhury et al. reasoned that a more acidic α-NH may increase the rate of dehydration and ring-closing to diazaborine products after initial imine formation. They realized their hypothesis by using sulfonyl hydrazides (**88**) as coupling partners for diazaborine labeling (Fig. 18B) [83]. Just as their semicarbazide analogs, the sulfonyl hydrazide formed stable adducts in aqueous buffer (pH = 7.4) at room temperature within minutes. The sulfonyl diazaborine products appeared thermodynamically more stable compared to their semicarbazide analogs, which was supported by exchange experiments and DFT calculations. Application of this method was demonstrated by fluorescent labeling and conjugation of KLA (an apoptosis-inducing peptide) to lanreotide for imaging and killing cancer cells. While semicarbazone formation was described as irreversible, Zegota et al. identified a salicylhydroxamate functional handle (**91**) for pH-dependent reversible binding to boronic acids **85** (Fig. 18C) [84]. The utility of this reaction was demonstrated by fluorescent labeling of boronic acid-modified lysozyme at pH 8 followed by release of the label upon acidification.

Eising et al. have identified vinylboronic acids (**93**) as potent dienophiles for tetrazine ligation (Fig. 18D) [85]. The authors showed that the use of a vinylboronic acid substantially increased the reaction rate compared to the corresponding olefins. Further, the reaction rate was not only enhanced by rendering the olefin more electron rich, but also by the 2-pyridyl substitution on tetrazines (**94**) via coordination to boron’s vacant p-orbital assisting in the ligation reaction. Later, the authors followed up with a detailed investigation of this coordination effect supported by DFT studies. They found ortho-phenol substituted tetrazines as even more reactive, vinylboronic acid-selective and more stable dienophile coupling partners [86]. Interestingly, IEDDA reaction was not followed by loss of hydrogen furnishing an aromatic product, rather, it was followed by protodeborylation yielding a stable dihydropyridazine adduct. The utility of this new bioconjugation system was shown by successful installation of a vinylboronic acid synthon to HAS followed by ligation with a BODIPY-functionalized bipyridyltetrazine in aqueous buffer (pH = 7.4) at room temperature [85]. The authors were furthermore able to use this bioconjugation system for fluorescent labeling of proteasome in living cells [87].

### New Cyclic Alkyne Derivatives for SPAAC

Strain-promoted azide-alkyne cycloadditions (SPAAC) rely on highly strained medium rings bearing an endocyclic alkyne functional group. Strain in these systems can be increased by the inclusion of sp^2^-hybridized centers or small rings in the cycloalkyne structure. The increased strain leads not only to higher reaction rates, but also to destabilization, resulting in undesired side reactivity with e.g. cysteine residues [88]. Electronic activation, in contrast, enables high reaction rates, while simultaneously decreasing ring strain. Exocyclic heteroatom substitution as well as inclusion of heteroatoms at the propargylic position in the cycloalkyne has proven to be effective in electronic activation [89, 90].

**Fig. 19:**
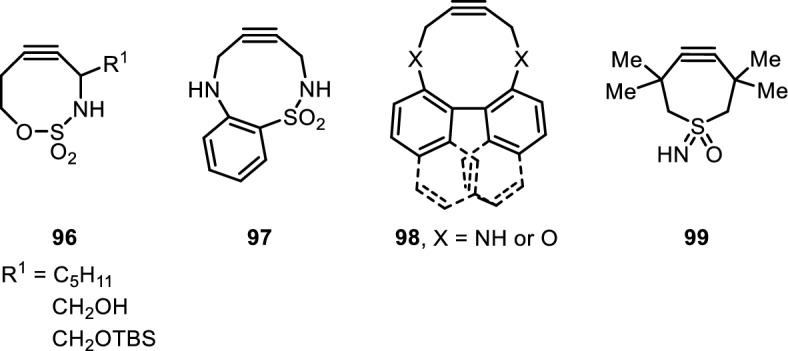
Selection of strained cycloalkyne derivatives for SPAAC reactions

Recently, the influence of heteroatom substitution at the homopropargylic position was investigated by Burke et al. [91]. They observed that propargylic and homopropargylic heteroatom substitutions separated by a sulfonyl bridge (e.g. alkyne **96**) affect both the alkyne electronics and alkyne distortion via remote hybridization and stereoelectronic effects, thereby opening new paths for modulating strain (Fig. 19). Reaction rates were faster than solely electronically activated cyclooctynes and reached the same order of magnitude as alkynes relying on high ring strain. The utility of this new strained alkyne was demonstrated by biotin labeling of P19C RNase 1. The authors additionally made use of the sulfonamide nitrogen to install functional handles.

Cyclononynes **97** bearing two propargylic heteroatom substitutions were reported by Kaneda et al. (Fig. 19) [92]. Due to their design with two inherently different nitrogen atoms (sulfonamide- and aniline-type), they could sample electronic effects of both to tune the reactivity. Decreased reaction rates for SPAAC with benzyl azide were observed upon acetylation of the aniline nitrogen atom, whereas increased reaction rates were observed with Boc, Cbz and Ts groups on the sulfonamide nitrogen atom. The authors reasoned based on crystal structures that a transannular hydrogen bond from the aniline NH to the sulfonamide oxygen accelerated the SPAAC reactivity. Reactions were carried out exclusively in organic solvents, and it remains to be seen whether these reagents can be applied in aqueous buffer.

Harris et al. realized that propargylic heteroatom substitutions in medium rings could serve a dual purpose. As previously exploited, they can exert hyperconjugative effects on the alkyne, but can also be involved in conjugation with an arene. The *n*–π* conjugation was disrupted in twisted cyclodecyne **98** (Fig. 19), but was restored in the transition state as torsional strain is released [93]. This remote stabilization increased the cycloaddition reaction rate as compared to previously reported cyclononynes.

Weterings et al. developed strained tetramethylthiacycloheptyne sulfoximine **99** (TMTHSI, Fig. 19) [94]. TMTHSI accounted for a faster reaction rate than DBCO and endo-BCN and comparable to BARAC while having a lower LogP, potentially facilitating a better aqueous solubility. The incorporated sulfoximine could easily be diversified with various functional handles. The utility of this new alkyne was demonstrated by nano-particle functionalization of TMTHSI-diversified HER2-targeting peptide as well as construction of peptide oligonucleotide conjugates.

**Fig. 20:**
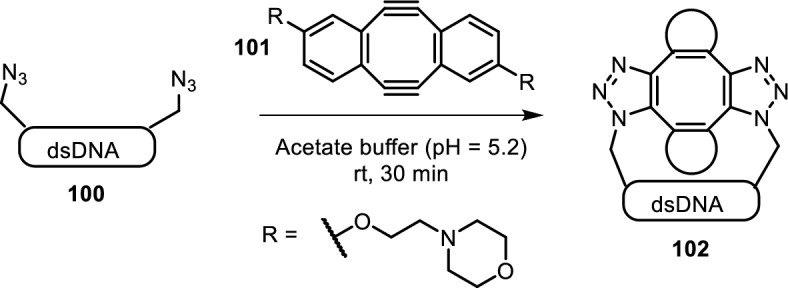
DiMOC **101** first interchelates dsDNA, followed by double SPAAC with azido-modified nucleotides

DNA interstrand cross-linking was achieved by Tera et al. This approach relied on incorporation of an azido nucleotide and modified cyclooctadiyne **101** (CODY, Fig. 20) [95]. The reported morpholine substitution, termed DiMOC, increased solubility compared to the parent CODY, while exhibiting low cytotoxicity. Interstrand cross-linking of azido nucleotides was achieved by first interchelation of DiMOC into the DNA duplex, followed by double SPAAC, resulting in highly toxic effects in cell viability assays. Application of this technique in vivo resulted in significant cell death indicating successful cross-linking of dsDNA. This technique may find potential application in chemotherapy.

### Tetrazine-Based Turn-On Probes

The development of bioconjugative fluorogenic reactions, i.e. reactions that yield fluorescent products from non-fluorescent starting materials, is challenging, especially in a bioconjugation context.

**Fig. 21:**
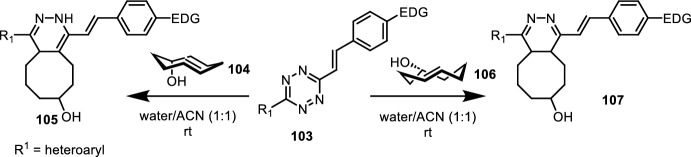
IEDDA with axial TCO **104** rapidly gives 1,4-dihydropyridazine fluorophore **105**. Reaction with equatorial TCO **106** yields 4,5-dihydropyridine **107** that only slowly tautomerizes to the 1,4-dihydropyridazine

Vasquez et al. and Siegl et al. found that tetrazine conjugations to TCO could yield the unprecedented fluorescent 1,4-dihydropyridazine product **105** (Fig. 21) [96]. They were able to tune the photophysical properties by substitution on the tetrazine. More interestingly, they observed that fluorescent products were only obtained for axial TCO **104** and not for equatorial TCO **106**. NMR and DFT calculations suggested that the initial Diels–Alder product of axial TCO 109 rapidly tautomerized to the fluorescent 1,4-dihydropyridazine product, while the Diels–Alder product of equatorial TCO **106** slowly tautomerized by water addition and elimination. By labeling of a short peptide sequence, they showed that treatment with axial TCO **104** yielded a fluorescent signal within a few minutes, while fluorescence was observed after a few hours for samples treated with equatorial TCO **106**. They further demonstrated the biocompatibility and cell permeability of axial TCO **104** by labeling of intracellular compartments (microtubules and mitochondria) in living cells. Building on the kinetic tautomerism, the very same group identified tetrazines that yield a 4,5-dihydrotetrazine fluorophore by reacting with equatorial TCO **106** [97]. As expected, the fluorescent signal decreased over time. The same fluorescent behavior was observed by labeling mitochondria and glycoconjugates in living cells.

**Fig. 22:**
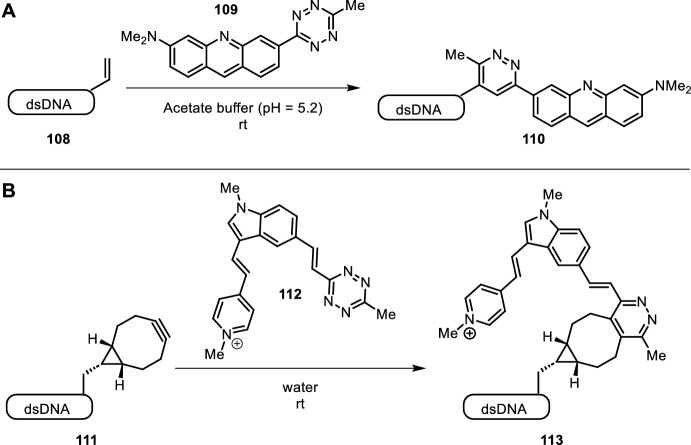
Dual functionality of tetrazine as a bioorthogonal IEDDA handle and fluorescent quencher for labeling of dnDNA utilized in PINK probe **109** (**A**) and compound **112** (**B**)

Loehr et al. reported a novel PINK probe (**109**) for vinyl-functionalized dsDNA, overcoming issues with high background fluorescence of unreacted labeling reagents of previously reported methods by inherent design (Fig. 22A) [98]. The PINK probe linked a tetrazine to a fluorescent dye. The tetrazine moiety does not only serve as a bioorthogonal handle for IEDDA reactions, but also as a fluorescence quencher. Interchelation into dsDNA followed by IEDDA with vinyl-substituted nucleotides effectively removed the quenching group and revealed the turn-on fluorescent probe. PINK showed no cytotoxic effects. The authors visualized DNA of HeLa cells tracking DNA synthesis and cell division.

Geng et al. also relied on the dual functionality of tetrazine as a bioorthogonal handle and fluorescent quencher (Fig. 22B) [99]. In addition to electronic quenching of fluorescence by tetrazine, interactions between dye **11** and DNA blocked depopulation pathways that are commonly initiated by twisting around the central bridge bonds. This approach yielded a two-factor fluorogeniticity with longer dsDNA exhibiting a greater reaction rate and turn-on rate than their ssDNA counterparts. In vivo DNA visualization was carried out in HeLa cells to demonstrate the utility of this method. Both authors highlight the ease of use, as it represents a mix-and-measure fluorogenic assay for DNA replication and tracking in living cells circumventing cell fixation and denaturation steps of previously applied methods.

### Beyond Tetrazines

**Fig. 23:**
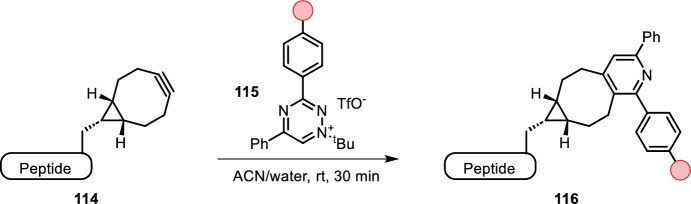
N1-alkylated triazinium salts **115** as IEDDA coupling partner for BCN

Slachtova et al. reported triazinium salts as new bioorthogonal reagents (Fig. 23, 115) [100]. They found that selective N1 alkylation enhanced reactivity by three orders of magnitude with endo- and exo-BCN compared to the corresponding non-alkylated 1,2,4-triazines. DFT calculations indicated a 10 kcal/mol difference in transition states, accounting for the enhanced reactivity. The ionic character of these compounds makes them more water-soluble and cell-permeable compared to the tetrazine analogs. N1 alkylation with a ^t^Bu substituent delivered the most reactive and most stable triazine reagent. Electron-donating groups decreased the reactivity, while electron-withdrawing groups exhibited higher reaction rates. Triazinium salts are selective for IEDDA with BCN, and react poorly with cyclooctynes including DBCO, which enables orthogonal labeling with e.g. azides. The authors synthesized triazinium salts bearing biotin, handles for amino acid functionalization (NHS ester, amines and arylpropionitrile), and fluorescent groups, which they were able to use in cell surface and intracellular labeling studies.

## Conclusion

Many diverse bioconjugation methodologies have emerged in recent years. In particular, the field of metal-mediated bioconjugations has seen the translation of commonly used transition-metal-mediated couplings from small molecules to biomolecules. The short reaction times and ease of diverse reagent synthesis make it appealing for application in biological systems. The field is still in its early stages of in vitro modification. Major challenges include the development of catalytic methods to reduce metal loading, site-selective modification and the removal of potentially toxic metals after successful protein modification. The discovery of new tags, such as the ATCUN motif, might aid in developing new site-selective modifications. While arylation has seen many examples in protein modification, alkylation, alkenylation and alkynylation are underexplored, and without doubt, new methods are on their way.

The number of different organic reagents targeting specific amino acids is impressive. Short and modular syntheses of the organic reagents facilitate their applicability in many contexts. An advantage of organic reagents is that primary amines (N-terminus and lysine) can be selectively targeted over more nucleophilic cysteine residues. However, only a rather small set of amino acids have been targeted so far for bioconjugation. Less nucleophilic sites including alcohols, carboxylic acids, primary amides, indole and imidazole have not yet been targeted. Although more challenging, the discovery of new bioconjugation methods and reagents can be expected in the near future.

The cyclopropenone bioorthogonal handle is a great addition to the existing toolkit and enables phosphine-based bioconjugation. It partially lifts the duality of azides, which are used not only in Staudinger ligations, but also commonly in cycloadditions.

The unique reactivity and coordination profile of boron render this element applicable in many bioconjugation reactions. The empty p-orbital serves a multitude of purposes including acceleration of reactions by coordination and selective adduct formation in boracycles. Importantly, boron-based product formation may be reversible, opening new avenues for applications of temporal bioconjugation (and release).

The development of reaction rate-tunable cyclic alkynes is aided by allylic and homoallylic heteroatom substitution combined with a better fundamental understanding of their reactivity. While most of these reagents are still in the first stages of development, applications in biological and bioconjugation context are already being developed.

The challenging discovery of fluorogenic turn-on probes might be advantageous for in vitro and in vivo labeling studies. In particular, the lack of background fluorescence and the two-factor fluorogenic signal might facilitate data analysis. Hence, we anticipate a variety of new applications for these techniques in the future.

## Data Availability

All data used in the review were retrieved from the cited references.

## Abbreviations

ATCUN: Amino terminal copper and nickel
BCN: Bicyclononyne
BDA: *cis*-2-Butene-1,4-dial
BSA: Bovine serum albumin
CODY: Cyclooctadiyne
CpO: Cyclopropenone
CuAAC: Copper-catalyzed alkyne azide cycloaddition
DBCO: Dibenzcyclooctyne
DFT: Density functional theory
FuTine: Furan thiol amine
IEDDA: Inverse electron demand Diels–Alder
PFAA: Pentafluoroaryl azide
PTAD: 4-Phenyl-3*H*-1,2,4-triazole-3,5(4*H*)-diones
SCO: *cis*-Cyclooctyne
SPAAC: Strain-promoted alkyne azide cycloaddition
SuFEx: Sulfur fluoride exchange
TCEP: Tris(2-carboxyethyl)phosphine
TCO: *trans*-Cyclooctene
TeTEx: Tetrazine thiol exchange
TMTHSI: Tetramethylthiacycloheptyne sulfoximine
